# 

**Published:** 1892-05

**Authors:** 


### BOOKS RECEIVED.

Transactions of the American Surgical Association, Volume the IX. Edited by J. Ewing Mears, M. D., Recorder of the Association. Philadelphia: Printed for the Association, and for sale by William J. Dornan. 1891.

The Principles and Practice of Medicine. Designed for the Use of Practitioners and Students of Medicine. By William Osler, M. D., Fellow of the Royal College of Physicians, London, Professor of Medicine in the Johns Hopkins University, and Physician-in-Chief of the Johns Hopkins Hospital, Baltimore. Formerly Professor of the Institutes of Medicine, McGill University, Montreal; and Professor of Clinic Medicine in the University of Pennsylvania, Philadelphia. New York: D. Appleton & Co. 1892.

The Pocket Pharmacy with Therapeutic Index. A Résumé of the Clinical Applications of Remedies Adapted to the Pocket-Case, for the

Treatment of Emergencies and Acute Diseases. By John Aulde, M. D., Member of the American Medical Association, of the Medical Society of the State of Pennsylvania, of the Philadelphia County Medical Society, etc. New York: D. Appleton & Co. 1892.

Fourth Annual Meeting of the National Association of the Railway Surgeons. Held at Buffalo, N. Y., April 30 and May 1, 1891. With a Historical Sketch of the Association, List of Members, and other Matters Relating to the Association. Illustrated, with portraits of the officers of the association and others. Published by the *Railway Age and Northwestern Railroader*, Chicago, Ill.

The Mediterranean Shores of America; or, The Climatic, Physical, and Meteorological Conditions of Southern California. By P. C. Remondino, M. D., Member of the American Medical Association, of the American Public Health Association, of the State Board of Health of California; Vice-President of the California State Medical Society, and of the Southern California Medical Society. Illustrated with forty-five engravings and two double-page maps. In one handsome, royal octavo volume, 176 pages. Extra cloth, price, $1.25 net; cheaper edition, bound in paper, price, 75 cents net. Philadelphia: The F. A. Davis Co., publishers, 1231 Filbert St.

A Manual of Diseases of the Nervous System. By W. R. Gowers, M. D., F. R. C. P., F. R. S., Consulting Physician to University College Hospital; Physician to the National Hospital for the Paralyzed and Epileptic. Second edition, revised and enlarged. Volume I., Diseases of the Nerves and Spinal Cord. With 180 illustrations, including 370 figures. Philadelphia: P. Blakiston, Son & Co., 1012 Walnut street. 1892.

The Book of Prescriptions, containing upwards of 3,000 Prescriptions, collected from the practice of the most eminent physicians and surgeons, English and foreign, comprising also a compendious History of the Materia Medica, List of the Doses of all official or established preparations, and an index of Diseases and Remedies, by Henry Beasley. Seventh edition. Philadelphia: P. Blakiston, Son & Co., 1012 Walnut street. 1890.

Fifth Annual Report of the State Board of Health and Vital Statistics of the Commonwealth of Pennsylvania. Transmitted to the Governor, December 2, 1889. Harrisburgh: Edwin K. Myers, State Printer. 1891.

Sixth Annual Report of the State Board of Health and Vital Statistics of the Commonwealth of Pennsylvania. Transmitted to the Governor, December 1, 1890. Harrisburgh: Edwin K. Myers, State Printer. 1892.

Practical and Analytical Chemistry. A Complete Course in Chemical Analysis. By Henry Trimble, Ph. M., Professor of Analytical Chemistry in the Philadelphia College of Pharmacy. Fourth edition. With illustrations. Philadelphia: P. Blakiston, Son & Co., 1012 Walnut street. 1892.

International Clinics: A Quarterly of Clinical Lectures on Medicine, Surgery, Gynecology, Pediatrics, Neurology, Dermatology, Laryngology, Ophthalmology, and Otology. By Professors and Lecturers in the leading medical colleges of the United States and Great Britain and Canada. Edited by John M. Keating, M. D., Philadelphia, Consulting Physician for Diseases of Women to St. Agnes' Hospital, Philadelphia; Editor

Cyclopedia of the Diseases of Children. J. P. Crozer Griffith, M. D., Philadelphia, Clinical Professor of Diseases of Children in the University of Pennsylvania; Professor of Clinical Medicine in the Philadelphia Polyclinic. J. Mitchell Bruce, M. D., F. R. C. P., London, England, Physician and Lecturer on Therapeutics at the Charing Cross Hospital. David W. Finlay, M. D., F. R. C. P., London, England, Physician to the Middlesex Hospital, and to the Royal Hospital for Diseases of Chest; Lecturer on Clinical Medicine in the Middlesex Hospital Medical School. October. 1891. Philadelphia: J. B. Lippincott Co.

Evidences of the Communicability of Consumption. By G. A. Heron, M. D. (Glas.), Fellow of the Royal College of Physicians, of London; Physician to the City of London Hospital for Diseases of the Chest. London: Longmans, Green & Co., and New York, 15 E. Sixteenth street. 1890.

Bacteriological Diagnosis: Tabular Aids for Use in Practical Work. By James Eisenberg, Ph. D., M. D., Vienna. Translated and augmented with the permission of the author from the second German edition, by Norval H. Pierce, M, D., Surgeon to the Out-Door Department of the Michael Reese Hospital; Assistant to Surgical Clinic, College of Physicians and Surgeons, Chicago, Ill. Philadelphia and London: The F. A. Davis Co., Publishers. 1892.

Diseases of the Urinary Apparatus; Phlegmasic Affections. By John W. S. Gouley, M. D., Surgeon to Bellevue Hospital. New York: D. Appleton & Co. 1892.

A Text-Book of Nursing for the use of training schools, families, and private students. Compiled by Clara S. Weeks-Shaw. Second edition, revised and enlarged with illustrations. New York: D. Appleton & Co. 1892.

Drinking Water and Ice Supplies and their Relations to Health and Disease. By T. Michell Prudden, M. D., author of The Story of Bacteria; Dust and Its Dangers, etc. G. P. Putnam's Sons, New York, 27 W. Twenty-third street; London, 27 King street, Strand. The Knickerbocker Press. 1891.

Lectures on Tumors from a Clinical Standpoint. By. John B. Hamilton, M. D., LL.D., Professor of Principles of Surgery and Clinical Surgery, Rush Medical College, Chicago; Professor of Surgery, Chicago Polyclinic; Surgeon, formerly Supervising Surgeon-General, U. S. Marine Hospital Service; Surgeon to Presbyterian Hospital, Chicago; formerly Professor of Surgery in Georgetown University; Surgeon to Providence Hospital, etc., etc. For the use of the students. Second edition. Detroit, Mich.: George S. Davis. 1892.

Transactions of the Medical and Chirurgical Faculty of the State of Maryland. Semi-annual session, held at Cambridge, Md., November, 1890. Ninety-third annual session, held at Baltimore, Md., April, 1891. Baltimore: Griffin, Curley & Co., printers, 202 East Baltimore street. 1891.

---

Notice to Contributors.—We are glad to receive contributions from every one who knows anything of interest to the profession. Articles designed for publication in the Journal should be handed in before the first day of the month. The Editors are not responsible for the views or opinions of contributors. All communications should be addressed to the Managing Editor, 284 Franklin St., Buffalo, N. Y.

### EFFECTIVE, PLEASANT AND SAFE ANTISEPTICS.

BY W. C. BAKER, M. D., Hummelstown, Pa.

IN THESE days of antisepsis but few physicians pretend to treat recent wounds of any character, or chronic abnormal conditions of any tissue in which the integrity of the parts is destroyed, without the efficient aid of one or more of the many antiseptics now in universal use. The conscientious, painstaking physician will not only use antiseptics in such cases, but he will also feel it obligatory to use such as are not only effective but also pleasant to the senses of the patient and safe as well. We all know how effective is iodoform. We also know how intolerable is its odor. The same may be said, relatively, of carbolic acid, of naphthol and many others, while we cannot shut our eyes to the dangers of effective solutions of meruric bi-chloride and the staining properties of potassa per manganas and argenti nitras.

My attention was recently called to two preparations of the Phenique Chemical Co., of St. Louis, Mo., viz: Campho-Phenique and Chloro-Phenique. I have had many opportunities of using both of these preparations, and can only say that I have used them with intense satisfaction. To me it is a satisfaction not to meet with the former salutation, "Doctor, you have been using that stinking iodoform." I can enter a house now and not be traced by my smell. I bind up a crushed finger with Campho-Phenique and remove the dressing a week afterward, with not a suspicion of fetid odor or formation of pus.

I treat a troublesome pruritis vulva with a solution (twenty-five per cent.) of Chloro-Phenique, and my grateful patient assures me that the itching has ceased. A troublesome, persistent, periodical, facial eczema which has resisted treatment by the formula of noted dermatologists, is "much better," and the bottle is brought in to be filled with Chloro-Phenique, which I tried experimentally. A fetid otorrhea is cured by ten days faithful syringing with a solution of Chloro-Phenique, and a long-standing leucorrhea is materially improved by cotton tampons soaked in a solution of Chloro-Phenique and inserted at bed time,

Thus varied and satisfactory has been my experience with Chloro-Phenique and its associate, and as a pleasant, very efficient and altogether desirable preparation I would be loath to part with either of them from my every day practice. So pleased have I been with them that I have felt constrained to write thus.

---

ACUTE HEPATITIS.—Sodium sulphate in the treatment of acute hepatitis—recommended by Thierfelder of Rostock, the best methods of medication with this salt is in the form of a natural water such as the water of Villacabras; as a therapeutic agent this water should not be classed with some of the so-called bitter waters of doubtful origin.

### AN INTERESTING OBSERVATION.

BY PROF. MARIUS ODIN, M. D., Nice, Chevalier of the Legion of Honor, etc.

MADAME DE G., of Austrian nationality, twenty-five years of age; married; no children; average constitution; lymphatic temperament.

I was struck, at first sight, with her pallor; her skin an the mucous membrane of her eyelids and lips were quite colorless.

This young woman complained of weakness and general atony, cephalagia, dizziness, vertigo, tendency to lipotynie, caused by sorrows, sitting up late at night, and generally depressing influences. There was gastralia, with alternate constipation and diarrhea. Menstruation was irregular, and an abundant leucorrhea was accompanied with gastralgic exacerbation. Her pulse was weak and depressible; there was a blowing sound with the first heart-beat; very accentuated in the carotids. On auscultation I found weak respiratory murmurs, much prolonged expiration; dry and jerking cough. There was insomnia and a tendency to night sweats.

Everything had been tried—tonics of all sorts, arsenic, iron, quinquinia could not be borne; hydrotherapeutics had given no results.

I prescribed for Madame de G. the Vin Mariani Erythroxylon Coca, from which I had had much satisfaction on several previous occasions, but which I had never used alone.

Want of appetite being one of the chief symptoms, and this keeping her general condition at a low ebb, I gave her a few doses of rhubarb, which, however, modified the situation but little. From that time I prescribed the Vin Mariani in doses of a claret glassful, morning and evening, a quarter of an hour before meals.

At the first doses she complained that it increased her dizziness. I assured her that this was a salutary and even necessary first effect of the medicine, and she consented, not without reluctance, to continue its use. At the end of eight days there was a notable amelioration. Appetite appeared, food was taken, and the digestive functions were becoming more regular—day by day. I then advised the patient to increase the dose by two more glasses per day, either after meals or between—whenever she had to undergo some exceptional fatigue.

Madame de G., who has since then resumed her daily occupations, tells me that, thanks to the medicament, taken at proper times, she can bear, without fatigue, long conversations and, at the same time, her vocal powers have acquired ampler development. At the end of a month's treatment, her state was most satisfactory; there remained a slight blowing with the first heart sound, which, however, was disappearing, and was not at all perceptible in the carotids any more.

This observation seemed to me very interesting and conclusive in this respect, viz., that it shows the action of Vin Mariani, when administered without any other medicament, and, what is no less interesting, it shows its useful effects upon the vocal organs—a fact first determined by the eminent specialist, Professor Charles Fauvel, who has given to it the name of Tensor of the Vocal Cords.—*Gazette de Therap.*

